# Vaccination practices of pediatric oncologists from eight states

**DOI:** 10.1186/s12913-023-10160-z

**Published:** 2023-11-07

**Authors:** Karely M. van Thiel Berghuijs, Heydon K. Kaddas, Echo L. Warner, Douglas B. Fair, Mark Fluchel, Elizabeth D. Knackstedt, Anupam Verma, Deanna Kepka, Adam L. Green, Andrew B. Smitherman, Lauren Draper, Rebecca H. Johnson, Anne C. Kirchhoff

**Affiliations:** 1 Cancer Control and Population Sciences, Huntsman Cancer Institute, Salt Lake City, UT 84112 USA; 2 University of Arizona Cancer Center, Tucson, AZ 85719 USA; 3 Department of Pediatrics, Division of Pediatric Hematology/Oncology, University of Utah, Salt Lake City, UT 84108 USA; 4 Primary Children’s Hospital, Intermountain Healthcare, Salt Lake City, UT 84113 USA; 5 Seattle Children’s Cancer and Blood Disorders Center, Seattle Children’s Hospital, Seattle, WA 98105 USA; 6 Division of Pediatric Infectious Diseases, Department of Pediatrics, University of Utah School of Medicine, Salt Lake City, UT 84113 USA; 7 Pediatric Specialists of Virginia, Center for Cancer and Blood Disorders, Fairfax, VA 22031 USA; 8 Center for Cancer and Blood Disorders, Division of Oncology, Children’s National Hospital, Washington DC, 20010 USA; 9 College of Nursing, University of Utah, Salt Lake City, UT 84112 USA; 10 Children’s Hospital of Colorado/University Colorado, Aurora, CO 80045 USA; 11 Lineberger Comprehensive Cancer Center, University of North Carolina, Chapel Hill, NC 27514 USA; 12 Saint Louis University, St. Louis, MO 63103 USA; 13 Mary Bridge Children’s Hospital, Tacoma, WA 98405 USA

**Keywords:** Immunization, Childhood, Survivorship care, Provider recommendation

## Abstract

**Background:**

Vaccinations are a vital part of routine childhood and adolescent preventive care. We sought to identify current oncology provider practices, barriers, and attitudes towards vaccinating childhood and adolescent cancer patients and survivors.

**Methods:**

We conducted a one-time online survey distributed from March-October 2018 to pediatric oncologists at nine institutions across the United States (*N* = 111, 68.8% participation rate). The survey included 32 items about vaccination practices, barriers to post-treatment vaccination, availability of vaccinations in oncology clinic, familiarity with vaccine guidelines, and attitudes toward vaccination responsibilities. Descriptive statistics were calculated in STATA 14.2.

**Results:**

Participants were 54.0% female and 82.9% white, with 12.6% specializing in Bone Marrow Transplants. Influenza was the most commonly resumed vaccine after treatment (7030%). About 50%-60% were familiar with vaccine guidelines for immunocompromised patients. More than half (62.7%) recommended that patients restart most immunizations 6 months to 1 year after chemotherapy. Common barriers to providers recommending vaccinations included not having previous vaccine records for patients (56.8%) or lacking time to ascertain which vaccines are needed (32.4%). Of participants, 66.7% stated that vaccination should be managed by primary care providers, but with guidance from oncologists.

**Conclusions:**

Many pediatric oncologists report being unfamiliar with vaccine guidelines for immunocompromised patients and almost all report barriers in supporting patients regarding vaccines after cancer treatment. Our findings show that further research and interventions are needed to help bridge oncology care and primary care regarding immunizations after treatment.

**Supplementary Information:**

The online version contains supplementary material available at 10.1186/s12913-023-10160-z.

## Background

Vaccinations are a vital part of routine childhood and adolescent preventive care. There are over 15,000 children and adolescents diagnosed with cancer each year [1]. Many pediatric cancers (31%) are diagnosed in children who are under the age of five, the same ages at which the majority of childhood vaccines are recommended [2]. Having cancer and undergoing treatment during childhood and adolescence may disrupt the receipt of recommended vaccinations. Additionally, certain treatment regimens (e.g., high dose steroids) can inhibit immune response to vaccines, while at the same time, cancer treatment can result in a loss of immunity against vaccine-preventable infections [3]. All of these factors can leave childhood and adolescent cancer patients and survivors vulnerable to vaccine-preventable infections [4, 5].

For providers caring for childhood and adolescent cancer patients, there are published guidelines concerning vaccinations [6]. While receiving cancer treatment, a yearly influenza vaccine is recommended for most cancer patients [7]. Regarding other vaccines, the Children’s Oncology Group (COG) guidelines indicate that children receiving cancer therapy should not receive “live vaccines” like MMR (measles, mumps, rubella) and recommend that parents “talk to (their) health care provider before (their) child receives any vaccines” [8]. Once cancer therapy is complete, vaccinations are particularly important for survivors, as studies have shown they are more susceptible to contracting vaccine-preventable diseases (e.g. herpes zoster, pneumococcal disease, and influenza) than children without cancer [9–11]. The Infectious Disease Society of America (IDSA) Clinical Practice Guidelines for Vaccination of the Immunocompromised Host generally recommend that patients who have completed cancer treatment receive needed routine or catch-up vaccines when immunological recovery is complete (typically 3–6 months off therapy, depending upon therapies received) [12].

Despite these guidelines, studies demonstrate variation in approaches related to vaccinations and pediatric oncology, although research has been limited [13, 14]. Likewise, there is little known about how pediatric oncologists interact with caregivers (typically parents) and primary care providers (PCPs) about vaccines. The aim of our study was to identify practices, barriers (both individual and structural), and attitudes towards vaccination among United States (US) pediatric oncologists. We explored pediatric oncologists’ perceptions regarding their responsibilities for vaccinations during and after treatment, and what they indicate as the responsibilities of PCPs caring for survivors.

## Methods

This study was part of a larger project on vaccination after pediatric cancer, with the other two branches of this study including (1) a survey of caregivers of pediatric cancer patients and survivors, and (2) a survey of primary care providers who care for pediatric patients [15, 16]. For this aspect of the study, we surveyed pediatric oncologists from nine institutions across the US on their vaccination practices. Institutions were chosen to represent a variety of regions across the US: Dana-Farber/Boston Children’s Cancer Center, Boston, Massachusetts; New York University Langone Medical Center, New York City, New York; Mary Bridge Children’s Hospital, Tacoma, Washington; Seattle Children’s Hospital, Seattle, Washington; Primary Children’s Hospital, Salt Lake City, Utah; Children’s Hospital Colorado, Aurora, Colorado; Nationwide Children’s Hospital, Columbus, Ohio; Cardinal Glennon Children’s Hospital, St. Louis, Missouri; and North Carolina Children’s Hospital, Chapel Hill, North Carolina.

### Participants

Eligible participants included pediatric oncologists (attendings and fellows), currently seeing patients at one of the nine institutions. The nine institutions were selected due to preexisting research relationships with the desire to survey oncologists across a variety of geographic locations. The eligibility criteria specifically included attendings and fellows to obtain a broad sample of participants with differing levels of experience.

### Procedures

After the study team received approvals from leadership at each institution, potential participants were predominantly contacted through e-mail, although we did follow-up via mail with a subset of non-respondents to improve participation. Eligible participants completed the informed consent process and were enrolled in the study from March 2018 to October 2018. Of 165 eligible oncologists, 112 were consented and completed surveys (participation rate of 67.8%). One participant was later found to be ineligible, which left 111 completed surveys. We collected and stored survey data electronically in REDCap and input responses from the mailed hard copies into REDCap. Participants had the option to receive a $10 gift card for participation after completing the survey.

### Survey design

The survey was designed based on literature review and input from pediatric oncologists and a pediatric infectious disease specialist. This team met over several months to create and revise the survey items; due to little published on this topic, most survey items were developed by the research team rather than adapted from existing measures. Following this step, the draft survey was reviewed both by oncologists and primary care providers (PCPs) to provide feedback on the item wording and areas for revision. The survey was then reviewed by the Director of the University of Utah’s Clinical and Translational Sciences Institute's Survey Design and Measurement Core. The final survey included 32 items with the following domains: vaccine recommendations and strategies, barriers to post-treatment vaccination, availability of vaccinations in oncology clinic, familiarity with vaccine guidelines, and perceptions toward vaccination responsibilities. Within the survey, “cancer treatment” was defined for participants as, “any chemotherapy/medicine, surgery or radiation given for purposes of treating cancer”; "off-therapy patients" were those not currently receiving any of the described treatments.

### Survey domains

Participants were asked to indicate which vaccines (if any) were currently provided in their clinics and their vaccine recommendation strategies for both general pediatric cancer patients and for bone marrow transplant (BMT) patients, both during treatment and after treatment. Questions were asked regarding their knowledge of common vaccine guidelines for immunocompromised patients (Centers for Disease Control and Prevention's [CDC] Advisory Committee on Immunization Practices [ACIP] and the IDSA Clinical Practice Guidelines for the Immunocompromised Host) [12, 17]. Participants identified vaccination barriers experienced within their clinic (e.g. lack of time to administer or discuss, no records of previous vaccines), perceived family barriers (e.g. fear of adverse side effects, anti-vaccination beliefs), and what role they see PCPs playing in vaccinations after cancer treatment. Finally, participants were asked to describe in open-ended responses the types of services that should be provided by pediatric oncologists and PCPs after cancer treatment is complete. A copy of the survey can be found as supplementary material.

Oncologist demographic factors included gender, race, location of clinical practice, years since completing fellowship or fellowship status, and BMT specialty.

### Statistical analysis

Descriptive statistics were used to summarize the count and percentages of responses for each item. The majority of items were binary variables indicating endorsement of a topic. For a Likert response item indicating who should be responsible for vaccination in survivorship, we collapsed responses into three categories: agree/strongly agree, neither, and disagree/strongly disagree. We report differences in responses among the BMT providers as relevant.

For “Other” write-in responses, we grouped similar responses into four categories: 1) vaccine strategies, 2) barriers to vaccination, 3) PCP roles in vaccination after cancer, and 4) pediatric oncologist roles in vaccination after cancer. Illustrative quotes are highlighted in the text to provide further context to these topics. Data were managed and analyzed in STATA 14.2.

## Results

### Pediatric oncologist characteristics and clinic characteristics

A total of 111 pediatric oncologists representing nine medical centers in eight states participated in the survey. Participants were 54.1% female, 82.9% were white, and approximately 35% were ≤ 4 years from completing their fellowship (Table 1). Of the participants, 12.6% specialized in BMT. A total of 90.1% of participants stated that their clinic can, and does administer some vaccines, but only 63.1% stated that their clinic stocked vaccines.

**Table 1:** Demographic characteristics of pediatric oncologists and clinics

*N* = 111	N	%
Gender		
Male	51	45.9
Female	60	54.1
Race		
White	92	82.9
Asian	12	10.8
Other/Prefer Not to Answer	7	6.3
Ethnicity		
Hispanic	7	6.3
Non-Hispanic	104	93.7
State of Clinic		
Colorado	21	18.9
Massachusetts	6	5.4
Missouri	5	4.5
North Carolina	11	9.9
New York	9	8.1
Ohio	17	15.3
Utah	19	17.1
Washington	23	20.7
Fellowship status or years in practice		
Fellowship	4	3.6
0–4 years	39	35.1
5–9 years	21	18.9
10–14 years	15	13.5
15–19 years	11	9.9
20 + years	21	18.9
Bone Marrow Specialty^a^		
Yes	14	12.6
No	84	75.6
Clinic administers some vaccines^b^		
Yes	100	90.1
No	10	9.0
Clinic stock vaccines		
Yes	70	63.1
No	41	36.9

^a^Missing responses: *n* = 3

^b^Missing responses: *n* = 1

### Pediatric oncologists’ knowledge of vaccine guidelines and practices

Participants were asked about their familiarity of vaccine guidelines and practices (Table 2). The percent reporting familiarity with common guidelines on immunizations for immunocompromised patients was 62.2% for ACIP and 52.3% for IDSA. The majority of participants (88.3%) indicated that the only vaccine they recommend to cancer patients during treatment is the influenza vaccine, but 7.2% also recommended other vaccines during treatment. Of participants, 43.2% recommended household members have all vaccines and 48.7% recommended inactivated vaccines only.

**Table 2:** Pediatric oncologists’ vaccine guideline familiarity, recommendation practices, and strategies

*N* = 111	N	%
**Guideline Familiarity**^a^		
Advisory Committee on Immunization Practices (ACIP)	69	62.2
IDSA Clinical Practice Guidelines for the Immunocompromised Host	58	52.3
**General Vaccination Practices On and Off-Therapy**		
Recommend patients receive vaccinations while on therapy		
Influenza Only	98	88.3
Yes	8	7.2
No	4	3.6
Missing	1	0.9
Recommend vaccines for household members during therapy		
All vaccines	48	43.2
Inactivated vaccines only	54	48.7
Other	5	4.5
Missing	4	3.6
Recommend age appropriate vaccines to off-therapy patients		
All of the time	90	81.1
Most of the time	17	15.3
Some of the time	2	1.8
Missing	2	1.8
Recommend time after end of chemotherapy before vaccination		
Less than 3 months	1	0.9
3 to 6 months	26	23.4
6 months to 1 year	69	62.7
More than 1 year	3	2.7
Other	10	9.0
Missing	2	1.8
How often do patients comply with vaccine recommendations after therapy		
Always	5	4.5
Often	78	70.3
Sometimes	23	20.7
I don’t know	3	2.7
Missing	2	1.8
**Strategies for Vaccination after Treatment Ends**^a^		
Continue with regular vaccination schedule where it stopped	62	55.9
Vaccinate after assessing titer levels	29	26.1
Repeat full schedule	13	11.7
Booster dose regardless of previous vaccination	10	9.0
Other vaccination strategy	5	4.0

^a^Endorsed these items; may not sum to *N* = 111 as providers may not endorse any or could endorse more than one

For off-therapy patients, 81.1% of participants recommended age-appropriate vaccinations ‘all of the time’. The majority reported generally recommending that vaccines restart 6 months to 1 year after the end of chemotherapy (62.7%) whereas 23.4% recommended 3 to 6 months. Participants indicated 70.3% of the time that patients were “often” adherent with their vaccine recommendations. Among the 16 BMT specialists (data not shown in tables), 93.3% recommend age-appropriate vaccinations ‘all of the time’ for off-therapy patients, typically to restart 6 months to 1 year after chemotherapy (46.7%).

Regarding general strategies for vaccination after treatment, 55.9% recommended patients resume their regular vaccine schedule, 26.1% recommended checking titers prior to resuming vaccinations, 11.7% recommended repeating the full schedule, and 9.0% recommended providing booster doses (Table 2). Participants resumed influenza vaccines the most (70.3) of all vaccines, shown in the supplementary table (see Additional file 1), which displays specific practices by vaccine type. There were no differences for BMT providers.

### Pediatric oncologists’ reported barriers to providing vaccinations

In Table 3, participants indicated common provider/clinic and family barriers to vaccination after cancer treatment. Provider/clinic barriers included not having previous vaccine records for their patients (56.8%) and oncologists’ lack of time to determine which vaccines patients need (32.4%). Other common concerns were time barriers for discussing vaccines, ordering vaccines, and administering vaccines.

**Table 3:** Pediatric oncologists' report of provider/clinic and family barriers to vaccination after cancer treatment

*N* = 111	N	%
**Provider/Clinic Barriers**^a^		
Often don't have previous vaccine records	63	56.8
Lack of time to determine vaccines patients need	36	32.4
Lack of time during visit to discuss vaccinations	20	18.0
Lack of time for ordering from pharmacy	18	16.2
Lack of time during visit to administer vaccinations	13	11.7
Lack of time during visit to do titer testing	8	7.2
Unsure of the need for vaccination	8	7.2
Personal fear of side effects or adverse effects	1	0.9
Unconvinced about the efficacy of vaccines	0	0.0
Other	11	9.9
*Have not experienced clinic barriers*	*8*	*7.2*
**Family Barriers**^a^		
Don't believe in vaccinations	66	59.5
Parent fear of side effects or adverse effects	49	44.1
Parents are concerned about patients’ immune system	46	41.4
Parents unsure of the need for vaccination	29	26.1
Patients fear of vaccination	22	19.8
Fear vaccines may increase the risk of relapse/new cancer	9	8.1
Other	3	2.7
*Have not experienced family barriers*	*16*	*14.4*

^a^Providers selected all that applied and values may sum to more than *N* = 111

For family barriers, 59.5% of oncologists reported that some patients’ families did not believe in vaccinations, followed by parent fear of side effects (44.1%), concerns about their child’s immune system after cancer (41.4%), and feeling unsure about the need for vaccines (26.1%). Write-in responses indicated that oncologists recommend that patients receive vaccines with their PCPs, but that they do not track whether the patient or their caregiver followed through with the recommendation. There were no differences for BMT providers regarding barriers.

### Pediatric oncologists’ perceptions of primary care providers’ roles in vaccine management

**Fig. 1:**
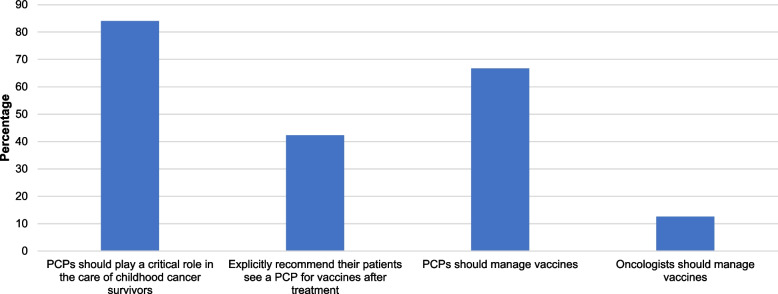
Perceptions of primary care provider (PCP) roles in vaccine management. Participants could select more than one response; as such, the total percentage exceeds 100%

We also asked participants their views of primary care and vaccine management (Fig. 1). Over 84% of participants reported that they feel that PCPs should play a critical role in the care of childhood cancer survivors. However, only 42.3% reported explicitly recommending their patients see a PCP for vaccines after treatment. When removing participants who believe vaccinations should be managed by an oncologist, the percentage of providers referring patients to see a PCP for vaccines after treatment increased slightly to 48.3% (not shown in figure). When asked who should administer vaccines, 66.7% of pediatric oncologists stated that vaccination should be managed by PCPs compared to 12.6% saying this was the role of oncologists (Fig. 1).

### Perceived health care roles

We asked two open-ended questions to understand participants’ views on health care responsibilities after cancer treatment. The first question read, “What aspects of healthcare provided after cancer treatment do you think the treatment center/oncologist should be responsible for?” The second question read, “What aspects of healthcare provided after cancer treatment do you think primary care providers should be responsible for?”.

Concerning pediatric oncologists’ role in the care of cancer survivors, three responsibilities were repeatedly mentioned: 1) Monitor for cancer recurrence, 2) Conduct surveillance for cancer treatment late effects, and 3) Communicate with the PCPs about cancer treatment and follow-up care. Pediatric oncologists also mentioned that it is their responsibility to ensure a safe transition of care, provide guidance on proper follow-up care after cancer treatment, and provide written recommendations (like a survivorship care plan) to their patients’ PCPs. Some respondents that mentioned vaccinations specifically, *“We should be responsible for ‘return to normal’ which in my view includes vaccination.”*

For PCP responsibilities, most participants stated that PCPs are responsible for the routine healthcare, including vaccinations. Often the participant would then mention that PCPs should only vaccinate after the oncology team recommended it: *“Vaccination would be great, if [PCPs] agree to do it per the recommendations of the specialist.”* Another common topic was continual communication between the oncology team and the PCP: *“We actively work to include the primary provider in our management plan and provide significant resources to minimize disruption of normal developmental trajectory. Therefore we feel that the primary provider is an essential part of ‘survivorship’.”* Some participants explained that their patients needed to transition back to a PCP to lower the costs of routine healthcare and to simplify the record keeping for items like vaccinations.

## Discussion

In this large sample of pediatric oncologists from nine institutions, we found different approaches regarding vaccination. Approximately half of participants were familiar with ACIP and IDSA vaccination guidelines for immunocompromised patients. Participants also described both clinic (e.g., lack of access to previous vaccine records or time to ascertain needed vaccines) and family (e.g., anti-vaccine or worries about side effects) barriers. While most participants believe that PCPs should play a large role in managing post-treatment care including vaccines, there may be a disconnect between these beliefs and the actual recommendations given to patients, as less than half of participants described explicitly recommending to their patients that they should transition back to a PCP for vaccinations. Our results point to a need for improving guidance and transition after cancer treatment for both providers and patients to ensure standardized approaches regarding vaccinations for pediatric cancer survivors.

Almost two-thirds of participants in our sample generally recommend that patients restart vaccines 6 months to 1 one year after chemotherapy, which differs from IDSA’s recommendations of restarting 3 to 6 months off therapy, depending upon therapies received [12]. Half of participants recommend that their patients simply continue with their vaccine schedule where it stopped before cancer treatment, while fewer recommended assessing titer levels to guide their vaccination plan, repeating the full vaccination schedule, or providing booster doses regardless of previous vaccination. In addition to finding variation in revaccination practice, our results echo an earlier report of 67 Children’s Oncology Group investigators that found most did not routinely assess vaccine status after treatment [18]. This COG study also reported that most clinics do not have provider guidelines on immunizations. Together, these findings demonstrate a critical need to ensure that oncology providers caring for survivors have appropriate guidance on vaccines after treatment.

In our survey, we assessed how oncologists perceive the role of oncology and primary care for survivorship care. Participants reported that oncologists and PCPs should communicate with one another about the vaccination strategy that should be used for their mutual patient. Several participants reported that PCPs should only provide vaccines upon recommendation from the oncologist. In a previous pilot study regarding survivorship care plans and care transitions, we found that pediatric oncology providers and primary care providers are not often in contact with one another about their mutual patient [19]. At the same time, the transition into survivorship care and primary care – and for some patients into adult care – is often fragmented, not standardized, and can be difficult for patients to navigate [20–22]. With these and other unnamed barriers, some childhood and adolescent cancer survivors may not have access to vaccinations for years after treatment has ended. Identifying these barriers are critical to establish a standard plan or flow of medical responsibility for missed vaccinations and re-immunization after therapy for cancer survivors.

Additional barriers to vaccination management after cancer treatment reported by pediatric oncologists included a lack of access to previous medical records and a lack of time to determine what vaccines a patient needed. In fact, over half of participants reported not having vaccine records. A third lacked time to determine what vaccines patients need. Ensuring that pediatric oncologists have access to patient medical records early in the treatment process could allow adequate time to plan recommendations, including vaccination, after treatment. Many electronic health record systems can pull vaccination records from state data through Meaningful Use Guidelines, which have been shown to improve immunization status in pediatrics [23]. Having this up-to-date information would help pediatric oncologists make recommendations to their patients and communicate with PCPs, and potentially address some of the time issues also reported.

Participants reported several potential family barriers regarding vaccination after cancer treatment that would be amendable to intervention including the most common concerns including (1) family vaccine skepticism and/or (2) fear of side effects. From the data gathered in this study, we are not able to distinguish if the skepticism and fear the pediatric oncologists noticed in families was general to vaccines or if the skepticism and fear increased due to their child’s cancer diagnosis. In an earlier study from our team that focused on caregivers (largely parents) of childhood cancer survivors, participants responded with relatively few worries about vaccine safety after cancer. This may imply that the interactions with families who are vaccine hesitant, skeptical or fearful of side effects may have been particularly poignant for pediatric oncologists in this sample even if these types of interactions are more rare than common. What was a primary driver of concerns about vaccines in our earlier study was that approximately one-third of caregivers reported not knowing which vaccinations to get, and that one-third of caregivers reported not discussing restarting their child's vaccinations with their cancer care team [24]. Report of a previous pediatric oncologist discussion about immunizations led to a much higher intention to vaccinate their child after cancer treatment [24], demonstrating a clear need for pediatric oncologists to address parent knowledge gaps on vaccines after cancer.

This study has certain limitations. As this was conducted prior to the COVID-19 pandemic, we did not address COVID-19 vaccination. The number of pediatric oncology locations in our study was small; however, our sample reflects geographic diversity across the US. Because of the small number of participants at each site, we were unable to examine patterns in responses. In addition, our assessment of vaccine practices was general. We were unable to ask specific detailed questions on pediatric oncologist behaviors related to vaccine efficacy and safety after certain treatment regimens. However, based on our findings and earlier reports demonstrating variability in vaccine practices after treatment, our study provides direction to future assessments to gather data that are more granular on this topic. All data reported in this manuscript are self-reported; future studies on this topic should include direct assessment of clinic vaccination practices to identify specific targets for improvement. Also, the survey used for this study is not a validated tool; it was created by the study team.

Finally, we did not design this study to ascertain differences between medical oncologists and BMT providers. There are standard post-transplant vaccination guidelines and for most BMT patients, the standard is to revaccinate after transplant. Thus, future work should investigate how BMT providers guide patients after treatment regarding vaccinations as those efforts could also inform general oncology practices. At the same time, evaluating practices of other providers, such as AAPs who are closely involved with survivorship care in many clinics, should be a focus of future research.

## Conclusion

Our findings demonstrate that support is needed to bridge oncology and primary care regarding immunizations after treatment [18, 25]. Following the end of treatment, pediatric oncologists should prioritize discussions with their patients and families regarding the importance of vaccinations and provide clear guidance to primary care providers on their recommendations. This is of upmost importance, as our earlier work clearly demonstrates that parents value oncologist recommendations as the expert in their child’s health [15, 16, 26]. At the same time, institutional guidance, as well as clinical guidelines, on vaccine practices are needed to help guide oncologists as participants reported different approaches with their patients. As vaccinations remain an essential and safe practice for most pediatric cancer survivors, it is of vital importance that childhood and adolescent cancer survivors receive vaccinations to reduce their risk of vaccine-preventable diseases.

### Supplementary Information

**Additional file 1.** Pediatric oncologist vaccination practices after cancer treatment by vaccination type.

## Data Availability

The data that support the findings of this study are available from the corresponding author upon reasonable request.

## Abbreviations

ACIP: Advisory committee on immunization practices
BMT: Bone marrow transplant/transplantation
COG: The children’s oncology group
IDSA: The infectious disease society of America
MMR: Measles, mumps, rubella
PCP: Primary care provider
