# Supplementary material for: Vaccination practices of pediatric oncologists from eight states
Source: BMC Health Serv Res. 2023 Nov 7;23:1215. doi: 10.1186/s12913-023-10160-z (PMC10629174; doi:10.1186/s12913-023-10160-z)
Supplement: Supplementary file 1 — Additional file 1. Pediatric oncologist vaccination practices after cancer treatment by vaccination type. [file 12913_2023_10160_MOESM1_ESM.docx]

**Additional file 1: Pediatric Oncologist Vaccination Practices after Cancer Treatment by Vaccination Type**

| **N=111** | Resume Appropriate Schedule | | Vaccinate for Missed Doses | | Extra Doses for Those Fully Vaccinated Before Diagnosis | | Order Titer Testing | | Never Recommend | |
| --- | --- | --- | --- | --- | --- | --- | --- | --- | --- | --- |
|  | **N** | **%** | **N** | **%** | **N** | **%** | **N** | **%** | **N** | **%** |
| Chicken Pox/Varicella (VAR) | 52 | 46.9 | 48 | 43.2 | 12 | 10.8 | 24 | 21.6 | 0 | 0.0 |
| Diphtheria, tetanus & pertussis (DTaP) | 54 | 48.7 | 51 | 46.0 | 18 | 16.2 | 13 | 11.7 | 0 | 0.0 |
| Haemophilus influenzae type b (Hib) | 59 | 53.2 | 47 | 42.3 | 16 | 14.4 | 8 | 7.2 | 2 | 1.8 |
| Hepatitis A (HepA) | 55 | 49.6 | 41 | 36.9 | 9 | 8.1 | 7 | 6.3 | 6 | 5.4 |
| Hepatitis B (HepB) | 54 | 48.7 | 42 | 37.8 | 14 | 12.6 | 13 | 11.7 | 2 | 1.8 |
| Human Papillomavirus (HPV) | 65 | 58.6 | 37 | 33.3 | 10 | 9.0 | 2 | 1.8 | 2 | 1.8 |
| Influenza (IIV) | 78 | 70.3 | 17 | 15.3 | 8 | 7.2 | 1 | 0.9 | 0 | 0.0 |
| Measles, mumps, rubella (MMR) | 53 | 47.8 | 47 | 42.3 | 14 | 12.6 | 15 | 13.5 | 0 | 0.0 |
| Meningococcal (Menveo, Menactra; MCV4) | 55 | 49.6 | 44 | 39.6 | 13 | 11.7 | 5 | 4.5 | 2 | 1.8 |
| Meningococcal B (MenB) | 51 | 46.0 | 34 | 30.6 | 8 | 7.2 | 3 | 2.7 | 10 | 9.0 |
| Pneumococcal conjugate (Prevnar) | 56 | 50.5 | 47 | 42.3 | 12 | 10.8 | 9 | 8.1 | 3 | 2.7 |
| Pneumococcal polysaccharide (Pneumovax; PPSV23) | 49 | 44.1 | 36 | 32.4 | 12 | 10.8 | 9 | 8.1 | 7 | 6.3 |
| Poliovirus (IPV) | 60 | 54.1 | 46 | 41.4 | 12 | 10.8 | 7 | 6.3 | 1 | 0.9 |
| Rotavirus (RV) | 57 | 51.4 | 23 | 20.7 | 4 | 3.6 | 1 | 0.9 | 20 | 18.0 |
| Tetanus, diphtheria, acellular pertussis (Tdap) | 58 | 52.3 | 44 | 39.6 | 11 | 9.9 | 11 | 9.9 | 1 | 0.9 |

Rows sum to >100% as participants could select more than 1 response
